# Grassroots: Leveraging mission-aligned developmental programs to enhance the academic health center

**DOI:** 10.1017/cts.2025.10100

**Published:** 2025-08-07

**Authors:** Jennifer A. Croker, Keith A. Jones, Anupam Agarwal, David T. Redden, Robert P. Kimberly

**Affiliations:** 1 Department of Medicine, University of Alabama at Birmingham, Birmingham, AL, USA; 2 UA Health Services Foundation and UAB Medicine, University of Alabama at Birmingham, Birmingham, AL, USA; 3 Heersink School of Medicine, University of Alabama at Birmingham, Birmingham, AL, USA; 4 Department of Biomedical Affairs & Research, Edward Via College of Osteopathic Medicine, Auburn, AL, USA

**Keywords:** Academic health center, faculty practice, strategic development, transdisciplinary collaboration, culture

## Abstract

Academic health centers (AHC) characterized by an integrated mission serving education, research and clinical care reflect these values in the institution’s vision, decision-making and culture. Embracing this strategy, the University of Alabama at Birmingham (UAB) created a novel, competitive funding initiative through its faculty practice with the Health Services Foundation General Endowment Fund (HSF-GEF). This partnership with the faculty practice leveraged faculty and staff creativity to inform and lead capacity-building and innovation in patient-oriented and laboratory research, clinical care development and education aligned with the best interests of the enterprise. Since 1996, the HSF-GEF has invested over $66M in 442 peer-reviewed proposals led by transdisciplinary teams representing strategic advances with strong potential to generate future extramural support, to improve healthcare delivery, to enhance research capacity and to promote active learning. Beyond financial return on investment, program evaluation revealed benefit on culture, collaboration, camaraderie and infrastructure. By engaging the broad workforce to articulate, select and implement projects, UAB has fostered a purpose-driven culture of collaboration within the AHC that thrives on broad representation, enthusiasm, and ingenuity as well as peer engagement across multiple schools in the academic community.

## Introduction

Strengths in the education and research missions represent differentiating features of top academic health centers (AHC). Missions that embrace an integrated vision, reflected in both leadership decisions and financial commitments that bridge domains, are a reflection of the institution’s implicit and explicit values and can have a major impact on culture [1,2]. In this perspective, the three principle components of most AHCs – the faculty practice, hospital, and medical school – are linked, creating the opportunity for a positive feedback loop that benefits patient care, research, and education. The clinical enterprise can contribute to the advancement of academic and research missions, facilitating cutting-edge discoveries that elevate access to evidence-based medicine and novel therapies for patients, creating a virtuous cycle. Pellegrini et al [2]., underscored the strength of the relationship with the faculty practice as “generally predictive of the vigor and success of the academic mission.”

Nonetheless, the individually prioritized missions of each academic health center component can create cultural gaps between research and clinical practice, which represent a potential barrier to the translation of evidence-based knowledge to healthcare application. Such divides may be rooted in different training and career development pathways between clinical providers and investigators. While specialized education for both is lengthy and highly demanding, these seemingly parallel pathways may struggle to converge and find common ground, leading to gaps in communication, respect and collaboration [3]. The contrast of goals between clinical care delivery and academic missions – that is, direct patient-centric benefit versus the creation of generalizable knowledge which may lead to patient benefit – also influence this divide. Overcoming this potential gap between healthcare delivery and research discovery relies on intentional efforts to nurture mutual appreciation of activities among clinicians, investigators, administrators and patients [4].

The medical center at the University of Alabama at Birmingham (UAB) has championed an organizational structure to transform patient care, advancing healthcare delivery to become the most highly ranked hospital in Alabama and one of the top AHCs in the United States. It currently provides health care services for more than 1.6 million patients annually, while training the next generation of both clinical and research professionals and advancing medical science through research [5]. The University of Alabama Health Services Foundation, P.C. (HSF) coordinates and delivers state-of-the-art patient care through a nonprofit physician group practice, consisting of over 1,500 faculty, fellows and advanced practice providers comprising a network of center- and community-based clinics that offer medical services in over 35 specialties.

### The General Endowment Fund program

**Figure 1.**
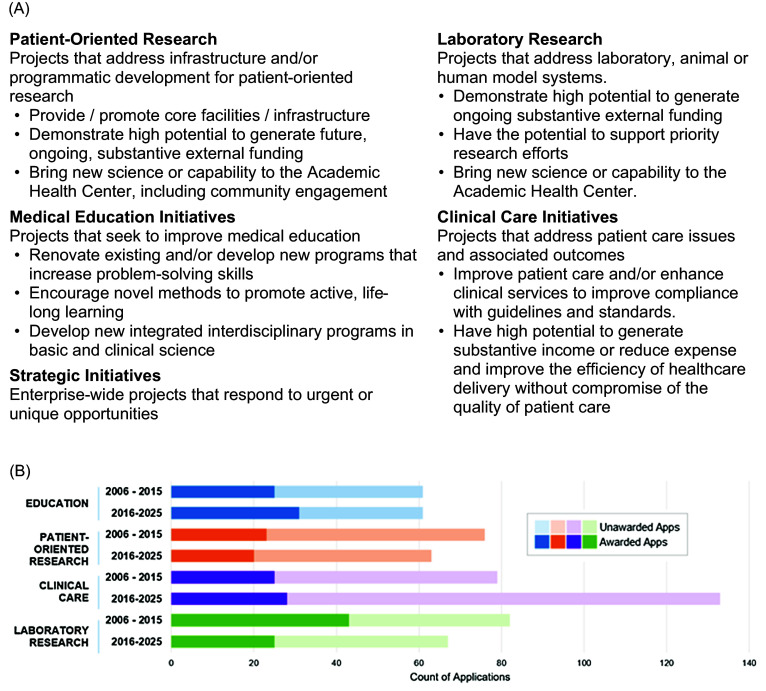
(A) Areas of emphasis of Health Services Foundation General Endowment Fund (HSF-GEF) grants. (B) Count of applications and awards over time by primary areas of emphasis.

In 1996, the HSF Board of Directors created an endowment fund to support a university-wide grants program for mission-aligned collaborative projects. The initiatives, enabled through this novel HSF General Endowment Fund (HSF-GEF), were designed to leverage the creativity of the faculty and staff and to catalyze capacity-building and innovation in mission-aligned areas of clinical care, education, patient-oriented and laboratory research consistent with the best interests of the enterprise. Through an annual competitive process, any faculty member in a department within the AHC may develop an idea, assemble a team, demonstrate multi-faceted support often with matching funds from stakeholders and submit a project proposal requesting seed funding for operational costs and/or new instrumentation in one of the four categories (Figure 1A).

HSF-GEF applications must be supported by the project lead’s department chair and include a project description (goals/benchmarks/benefits/details) and both management and sustainability plans. Successful applications typically create new capacities, enhance collaborative initiatives or amplify shared facilities rather than support single project-focused activities characteristic of many investigator-initiated pilot grant programs. These characteristics differentiate the HSF-GEF program from more traditional pilot & feasibility programs, which emphasize research projects, enable preliminary data, often target early stage investigators and foster individual career development. Designated review sub-committee chairs for each category (clinical care, education, patient-oriented, laboratory research) organize the primary assessment of HSF-GEF proposals. Each sub-committee is composed of thought leaders and peers across the institution to assess the merit of the proposal, the strengths of the team to lead the project and the importance to the enterprise. Committee participation is rotated annually to minimize conflicts of interest with that year’s application portfolio. Reviewers may not comment on applications if they are in the same department as the lead applicant, are a close collaborator or have contributed to a letter of support for the proposal. The committee chair determines if a reviewer with a perceived or real conflict should step away from discussion of a specific project. Reviewers assign each project an impact score on the 9-point NIH scale and offer written critiques. Each committee meets to discuss project attributes, assign scores and develop relative rankings for award recommendations. The four sub-group chairs meet with the HSF-GEF grant program director to integrate and prioritize project selections based on the availability of funds. These recommendations are presented to and discussed with the HSF-GEF Oversight Committee. The Committee is comprised of several department chairs and multiple board members who advise the HSF and co-chaired by a board member and the Dean of the School of Medicine. After discussion, the recommendations are provided to the full HSF Board of Directors for final consideration and award. Over the past ten years, the HSF-GEF has conferred 12–14 awards from a pool of approximately 36–40 applications annually. The requested budget for an HSF-GEF application ranges from $11,000 to $750,000 (average, $182,000), and awards range from $9,600 to $500,000 (average, $128,000). In some cases, review feedback was leveraged by collaborating groups to improve and resubmit a proposal, leading to subsequent awards 39% of the time.

Our team considered whether programmatic investments by the clinical practice in the research and academic missions, like the HSF-GEF effort, would have an influence on the culture of collaboration at the institution. Working with previous awardees, we used qualitative and quantitative assessments to explore the influence of this internal grant program.

## Methods for the assessment of impact

### Internal review board

In accordance with federal regulations, this project did not constitute human subjects research as defined under 45 CFR 46.102(d) and was determined by the IRB to be quality improvement and/or program evaluation.

### Quantitative survey and analysis

As part of a continuous quality improvement effort in the conduct of the HSF-GEF program, a convenience sample of 170 previous awardees with an active email address at the institution received an anonymous Qualtrics survey link. Thirteen HSF-GEF-related questions used a 5-point Likert scale and an open-ended feedback option to provide additional information. Responses were assessed by their level of agreement with specific benefits using sample proportions. An exact binomial test assessed whether the proportion agreeing differed from 50%. All tests were conducted using SAS 9.4 and utilized a Type I error rate of 0.05.

### Qualitative interviews

For contrast, a subset of previous HSF-GEF grant awardees who had changed institutions since the GEF award with forwarding contact information were engaged in a semi-structured interview to assess impressions of HSF-GEF program impact and influence. After 12 interviews, an emergence of recurring themes was observed with no new information appearing in the feedback (“saturation”).

### NIH funding analysis

Annual NIH budget history and NIH-funded projects at UAB for the years 1990 – 2024 were collected from the NIH RePORT Expenditures and Results (RePORTER) module (https://report.nih.gov/). UAB’s overall market share was defined as the quotient of UAB’s NIH funding / Total NIH dollars for a given year. The subset of UAB’s “P-series” and “U-series” grants was used to represent collaborative research program projects, research centers and cooperative agreements.

## Results

Since its inception, the HSF-GEF has invested nearly $66M in over 400 proposals to advance the mission of the academic health center. Current funding for this program is $1.5M/year. The number of applications in any given year has varied from 36–40, and, apart from clinical care proposals which have experienced a growth of more than 50% in the last 10 years, the number applications in each category has been relatively constant over time (Figure 1B; total application data were not available prior to 2005).

In the earliest phase of the GEF program, the GEF helped build laboratory research capacity in technology development and large equipment grants. In the first five years of the HSF-GEF Program (1996–2000), the emphasis on support for building laboratory capacity was reflected, in part, by UAB’s success in increasing NIH market share by >16% compared to the previous 5 years and in increasing NIH support for collaborative research program projects, research centers and cooperative agreements by 48%. Subsequently, these investments were broadened and have advanced strategic efforts in precision medicine, immunology and transplantation as well as neuroimaging, rather than specific funding mechanisms.

HSF-GEF investments in Medical Education have played a pivotal role in enhancing curricular development and implementation through skills laboratory upgrades and integration of stage-agnostic training platforms, including simulation, gamification, multimedia instruction and evaluation. These efforts were complemented by initiatives supporting health professional educator and leadership development to further the impact of training for new generations of learners and learning styles. Through its more recent support of Equal Access Birmingham, the GEF has integrated service-learning into medical education through a student-run, faculty-supervised, free clinic to provide care for the uninsured and underserved.

The Patient-Oriented Research portfolio has played a key role in building the clinical trial capacity [6] which has increased clinical trial expenditures, both industry and federal, by nearly four-fold to $138 million since the inception of the program in 2015. The POR portfolio also launched the development of UAB’s biorepository infrastructure which now supports over $25M in multi-institutional federal awards locally and nationally, including collaborative studies in multiple sclerosis, precision nutrition and chronic diseases. Having generated momentum in genomic medicine, UAB Medicine has earned a national reputation in precision health, evidenced in part by its role in major NIH initiatives, including *All of Us* and *Electronic Medical Records and Genomics (eMERGE)*. At the same time, targeted investments in the clinical enterprise have facilitated UAB Medicine becoming the leading health care provider in the state.

**Figure 2.**
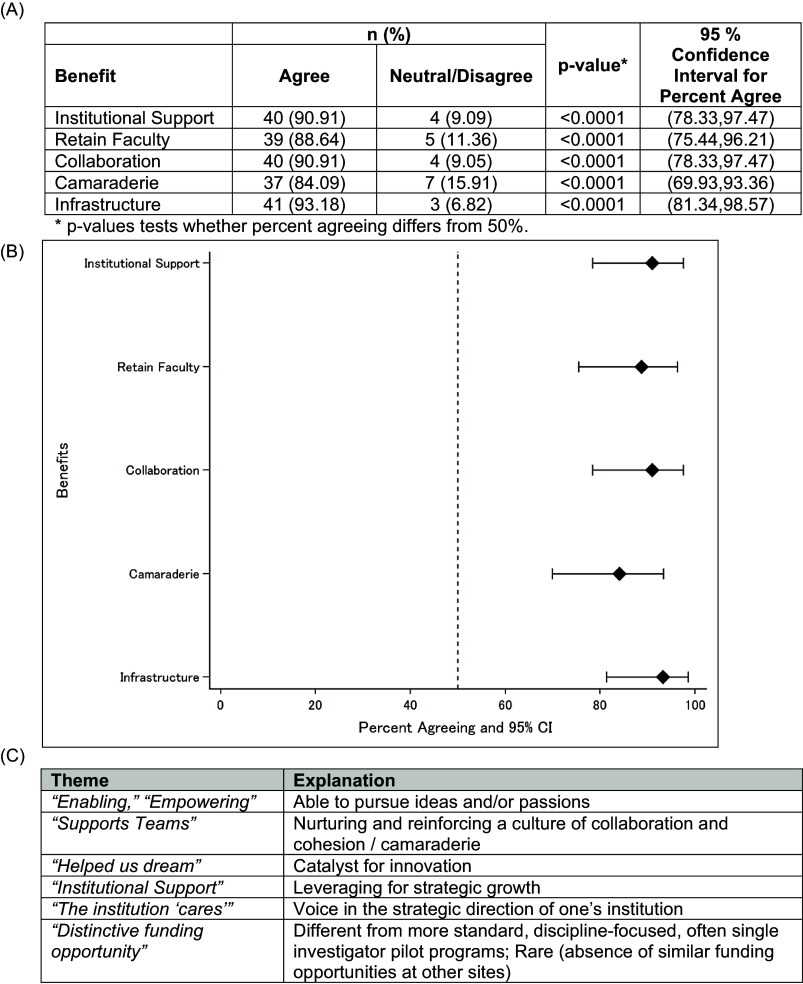
Quantitative and qualitative feedback. (A) Proportions of respondents to a quantitative survey still present at the institution agreeing/disagreeing with benefits. (B) A forest plot of each benefit depicting the pooled effect size (represented by diamonds) with horizontal lines indicating the 95% confidence interval for each individual category. (C) Recurrent themes from semi-structured interviews (qualitative survey) of awardees that have since left the institution (*n* = 12).

To understand any additional benefits attributable to the HSF-GEF program on the AHC beyond direct funding, a total of 44 (26%) HSF-GEF awardees currently at the institution responded to a survey that sought to evaluate perspectives of the program’s influence on infrastructure, camaraderie, collaborative culture and institutional support. Based on whether the proportion of respondents agreeing to a given benefit differed from 50% (Figure 2A**,** 2B), there was significant agreement (*p* < 0.0001) among responding HSF-GEF awardees that this program plays an important role in the culture of AHC ecosystem.

Qualitative feedback from key informants selected from among awardees in each of the four HSF-GEF programmatic areas who have since departed the institution provided additional insight into the influence of the HSF-GEF grant program to improve healthcare delivery, to enhance research capacity and to promote active learning (Figure 2C). In zoom-based interviews, using a structured discussion framework, several recurrent themes emerged: collaboration, cooperation, and cohesion; “empowering” with one colleague saying, “it helped us dream.” Every respondent commented on the uniqueness of the “bubble up” nature of the program and its enablement of faculty in pursuing collaborative goals.

## Programmatic impact

### Patient-oriented research (POR)

**Figure 3.**
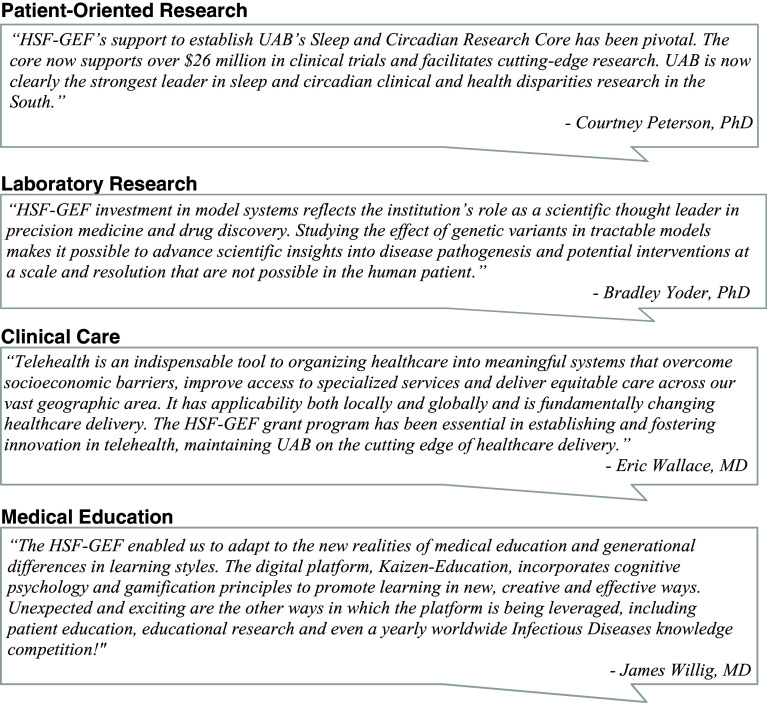
HSF-GEF impact statements.

The HSF-GEF POR program has played a vital role in clinical research innovation. For example, there is growing evidence linking disruptions of sleep or circadian health and a variety of disease conditions, including immune, psychologic, metabolic, cardiovascular and cognitive health [7]. An HSF-GEF award helped to establish a new multidisciplinary Sleep and Circadian Research Core integrated within the hospital footprint, permitting safe and rigorous studies related to the inter-connections of circadian rhythms, sleep, and multiple chronic health conditions. The first of its kind in the southeast United States and one of a few facilities nationally, this new facility is outfitted with state-of-the-art polysomnography and circadian rhythms research equipment, including capacity for frequent blood sampling throughout the night. It is strategically aligned with the institution’s primary clinical research unit and is immediately adjacent to specimen processing facilities and metabolic kitchen, all managed by the institution’s NIH Clinical and Translational Science Award (CTSA). This capacity development has led to a tripling of total NIH funding supporting circadian research, including multiple R01 grants exceeding $26M, as well as a flourishing sleep science community across multiple departments, schools and regional institutions (Figure 3).

### Laboratory research

During an era of rapid experimental innovation, including advances in genomics, neuroscience, cell biology and information science, the HSF-GEF Laboratory Research platform offered the means to compete at the cutting edge of science. In many cases, GEF investments represented matching support allowing the institution to successfully compete for more than 50 extramural infrastructure awards totaling more than $24M (e.g., NIH Shared Instrumentation Grants [S10]) to expand capacities in single cell analytics, microscopy/imaging, high performance computing and high-dimensional, high-throughput single cell ‘omics’. The latter has led to groundbreaking insights, including transcriptomic signatures associated with atherosclerosis [8] and rare diseases [9], characterization of tumor microenvironments [10], cell-specific pathogenicity in Parkinson Disease [11], comprehensive mapping of neurologic structures related to learning and memory in model systems [12] as well as immune cells in cystic kidney disease [13] and proof of principle of pharmacologic immunosuppression during porcine-to-human xenotransplantation [14].

HSF-GEF investments have also made a notable impact on the access to robust model systems for investigation, including a leading-edge zebrafish facility (Figure 3). Initial investments of approximately $500,000 led to over $6.8M in extramural funding within the first decade, resulting in an annualized return on investment exceeding 25%. The facility has promoted insights into metabolic homeostasis and the gut microbiome [15], preclinical investigation of novel therapeutics to treat disorders [16], genetic influences in epilepsy [17], and phenotypically variable ciliopathies [18]. It has also supported the generation of novel bioinformatics techniques for cross-species transcriptomic analysis [19] and led to UAB’s most recent NIH Director’s New Innovator award supported by the Common Fund.

### Clinical care development program

Over 80% of Alabama counties are considered rural and represent the home for nearly half of the state’s population. Seven of these counties do not have a hospital and 35 are maternity care deserts [20–22] Recognizing these challenges, UAB Medicine is committed to addressing health outcomes and improving healthcare delivery for all of the communities it serves.

The UAB Telehealth Program was established in 2015 with seed funds from the HSF-GEF and in partnership with the Health System and the Alabama Department of Public Health to create a statewide telehealth network of county health departments. This telehealth effort incorporated HIPAA compliant video technology and remote monitoring capabilities – as well as the requisite clinical and administrative workflows, data management strategies and informatics/information technology – to enable real-time, interactive communication and data exchange between the patient and physician or between physicians (Figure 3). In partnership with rural hospitals, UAB now provides telehealth inpatient care in critical care medicine, stroke and nephrology, leading to a 150% growth in patients receiving care at remote locations. The efforts begun in 2015 were vital in the response to COVID-19. Within one month of the pandemic’s start, UAB successfully transitioned over 74% of its outpatient clinic visits to telehealth. This pivot saved countless lives by enabling continuity of care while protecting patients and providers from exposure.

### Medical education

Through the HSF-GEF grant program, the Department of Medical Education established the Health Educators Academy (HEA), which serves as the hub for educational innovation for all faculty and departments in the Heersink School of Medicine. The HEA integrates faculty development and career mentorship of educators across all departments. As it inspires a culture of excellence in teaching, the HEA develops health professions educators by drawing on thought leaders from medicine, nursing, health professions, dentistry, public health and optometry. Among the first two cohorts of faculty interested in enhancing their skills, 50% received teaching awards, 33% were promoted and multiple scholarly papers were published in the field of health professions education [23]. Complementing its efforts in faculty development, the HSF-GEF played a seminal role in creating a career development pathway for research staff professionals. The Developing Emerging Administrative Leaders (DEAL) program takes a multidisciplinary approach in engaging promising UAB staff members in the essential components of administrative and management positions in an academic health center, including human resources, finance, research, education and clinical operations. The DEAL program has succeeded in nurturing a talented community of practice poised to support the institutional mission.

In partnership with the Center for Clinical and Translational Science (the CTSA Hub), the HSF-GEF Medical Education Initiative has similarly supported the development of adaptable workforce development platforms. Grounded in principles of adult learning theory and gamification, UAB informaticists created a web-based, self-directed, training platform called *Kaizen Education* (Japanese for “Continuous Improvement”) that offers an engaging alternative for delivering content to master competencies in any discipline [24]. In the process of learning, individuals and teams can compete for extrinsic (badges, scores, etc.) and intrinsic (collaboration, self-efficacy, etc.) rewards (Figure 3) [24]. Originally conceptualized to boost knowledge retention by internal medicine residents, Kaizen Education has engaged several thousand learners by providing health professional training (medical, nursing, dentistry), by assessing skills (biostatistics, clinical research) and by conveying concepts of rigor & reproducibility and good clinical practice [25–27]. It has also been piloted in patient education as a means to offer knowledge about diabetes, which was found to have a positive, “empowering” impact on a patient’s discussions with healthcare providers about their disease [28].

## Discussion

**Figure 4.**
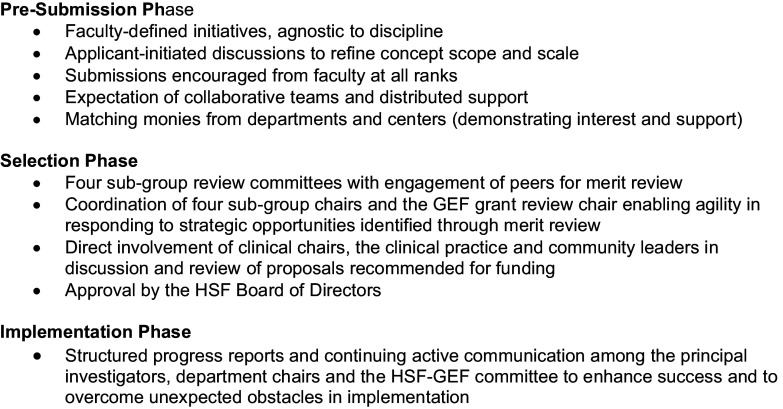
Characteristics of the HSF-GEF program.

By creating a faculty-driven grants program funded by its General Endowment Fund, the Health Services Foundation took intentional steps to engage the broad faculty in the development of enterprise-oriented opportunities in research, education and clinical care. Distinct from Center-driven pilot grant programs with their disciplinary focus on PI-led research projects, the HSF-GEF sought to advance innovative initiatives and capacities serving the faculty and the institution (Figure 4). To assess the impact of this program on the faculty and the broader AHC, we have examined its relationship to extramural funding and have collected feedback from both present and past awardees (Figure 2). Taken together, this evidence illustrates a positive impact of the HSF-GEF program that transcends the direct financial support of specific initiatives to affect perceptions of institutional support and culture throughout the AHC.

Like many intramural investment programs, the number, size and expense of ideas far exceeds the availability of funds. In hindsight, the HSF-GEF grant program’s design – an open call for faculty/staff-led concepts with modest, but without explicit, budgetary guidelines – may have fortuitously sidestepped two programmatic risks [29]. In terms of *intrinsic motivation*, large financial awards may actually dampen project enthusiasm among individuals and teams in an AHC environment by unintentionally “turning play [or professional passion] into work.” Applicants are empowered to specify the scope of their desired project and to recommend a true estimate of seed costs to accomplish their vision. Similarly, by avoiding institutionally-specified targets of what topics, activities or technologies the GEF wished to fund, the program mitigated the risk of top-down *extrinsic motivation*, known to hamper creativity, reduce buy-in, cripple innovation and sacrifice genuine commitment to ideas or projects [30].

Through its General Endowment Fund, the HSF has had a profound impact on the institution’s research ecosystem responsive to defined needs of investigators and teams. Reflecting the integrated mission that weaves the fabric of research, academic and clinical enterprise activities together, the HSF-GEF Program has played a significant role in creating a seamless bridge across missions. Over time, the HSF-GEF has become a cornerstone of the AHC as a catalyst in response to novel ideas and strategic opportunities identified and led by faculty and staff. Initial mixed-methods analysis supports a research development model characterized by engaged grassroots enthusiasm with the shared vision embraced by the UAB enterprise. As a result, the AHC at UAB has created a culture of collaboration and cohesion within which synergies in the academic, research and clinical care missions can thrive.

### Limitations

In a matrix organization, direct attribution of impact, whether on extramural funding or on organizational culture, is operationally complex. A program like the HSF-GEF can be both a driver of and a reflection of organizational strategy and culture. Nonetheless, review of the HSF-GEF portfolio clearly demonstrated its role in leveraging the institution’s success in multi-investigator, multidisciplinary grants, especially when NIH was putting particular emphasis on P- and U-series mechanisms. The experience from that impact reinforced the role of the GEF in advancing a culture of collaboration and cohesion, the seeds for which had been planted in research, education and clinical care. As with all surveys of past activities, the surveys of faculty currently at UAB and of faculty who had transitioned to other institutions were subject to respondent availability and to recall bias. The thematic consistency of comments in both online feedback and semi-structured interviews suggest a shared perception and experience with the process. The multi-decade perspective highlights the evolution of the program, the broadening of faculty interests and the realization of the program’s seminal impact on the institution’s commitment to a collaborative culture.
